# Perceived access and barriers to care among illicit drug users and hazardous drinkers: findings from the Seek, Test, Treat, and Retain data harmonization initiative (STTR)

**DOI:** 10.1186/s12889-018-5291-2

**Published:** 2018-03-20

**Authors:** Mika Matsuzaki, Quan M. Vu, Marya Gwadz, Joseph A. C. Delaney, Irene Kuo, Maria Esther Perez Trejo, William E. Cunningham, Chinazo O. Cunningham, Katerina Christopoulos

**Affiliations:** 1 Collaborative Health Studies Coordinating Center, University of Washington, Building 29, 6200 NE 74th St, Seattle, WA 98115 USA; 2 Johns Hopkins University, 615 N Wolfe St, Baltimore, MD 21205 USA; 3 Center for Drug Use and HIV Research, Rory Meyers College of Nursing, New York University, New York, NY 10003 USA; 4 George Washington University, Milken Institute School of Public Health, Department of Epidemiology and Biostatistics, 950 New Hampshire Avenue NW, Suite 500, Washington, DC 20052 USA; 5 Department of Medicine, Div GIM & HSR, Geffen School of Medicine, UCLA and Department of Health Policy and Management, Fielding School of Public Health, UCLA, Los Angeles, CA USA; 6 Albert Einstein College of Medicine & Montefiore Medical Center, 111 East 210th Street, Bronx, NY 10467 USA; 7 Division of HIV, ID and Global Medicine, Zuckerberg San Francisco General, University of California San Francisco, 995 Potrero Ave, 4th Floor, San Francisco, CA 94110 USA

**Keywords:** Barriers to care, HIV/AIDS, Substance use, Race/ethnicity, Social support

## Abstract

**Background:**

Illicit drug use (DU) and hazardous drinking (HD) among marginalized populations may be associated with greater barriers to care.

**Methods:**

We used baseline data on the participants of the Seek, Test, Treat, and Retain data harmonization initiative. DU includes use of any illicit drugs within the past 6 months. HD was defined as scores ≥8 for men and ≥ 7 for women on Alcohol Use Disorders Identification Test within the past 12 months. Social support scores were assigned by summing scores from individual questions related to social support. Two outcomes for multivariable regression models and mediation analysis were perceived access to care and perceived barriers to care scores, calculated from summated points from individual questions within each domain. All models were adjusted for age, gender, race/ethnicity, and social support and stratified by HIV status.

**Results:**

Among 1403 illicit drug users and 4984 non-drug users, the mean age was 39.6 ± 12.2 years old, 71% were male, 57% African Americans, and 39% Hispanic/Latinos. Over 25% reported difficulties in covering medical costs and finding transportation to health care facilities and greater proportions of drug users and hazardous drinkers reported these issues than non-DU/non-HD. In multivariable models, DU and HD were both independently associated with having greater barriers to care (β: 0.49 (95% confidence interval: 0.19 to 0.79) *p* < 0.01; 0.31 (0.18 to 0.45) < 0.01) in HIV-negative participants. Neither DU nor HD was strongly associated with barriers to care for HIV-positive participants. Social support was associated with better perceived access to care and fewer barriers to care in the HIV-negative participants.

**Conclusion:**

The current study found that financial burdens of care, logistical difficulties in accessing care, and low social support were common challenges among individuals using illicit drugs and/or drinking hazardously. Addressing structural barriers and strengthening social support may be important strategies to improve health care among marginalized populations, regardless of HIV status.

**Electronic supplementary material:**

The online version of this article (10.1186/s12889-018-5291-2) contains supplementary material, which is available to authorized users.

## Background

Socioeconomically marginalized populations are known to receive worse health care than more privileged populations in the United States. In the 2011 National Healthcare Quality and Disparities Reports, low-income populations reported worse access to care for 89% of access measures compared to high-income populations; African Americans and Hispanics/Latinos also had worse access to care than Whites for 32% and 63% of access measures, respectively [1]. Many different types of barriers to health care exist, including high costs of medical care, the need for insurance coverage, logistical concerns (*i.e.* travel time, clinic hours, means of transportation), and linguistic and cultural differences [2–5]. Low trust in health care systems and professionals is another common barrier to care, especially among racial/ethnic and sexual minority groups [6–8]. Specific types of barriers to care may vary within marginalized populations, for instance, by racial/ethnic background, HIV status, and substance use patterns.

Substance use is a pressing public health issue in the U.S. Recent national estimates showed that over 78 million people (29.8%) above age 12 years had used illicit drugs other than marijuana during their lifetime [9, 10] and over 15 million adults (6.2%) had alcohol use disorder [11]. The 2013 estimates suggested that those with lower socioeconomic status experienced greater illicit drug use; those without high school degrees had the highest rate (11.8%) of current illicit drug use while it was lowest among college graduates (6.7%); current illicit drug use was also higher among unemployed adults (18.2%) in comparison to full-time employees (9.1%) [12]. In contrast, the rate of alcohol use was higher among more highly educated adults; however, binge and heavy alcohol use was less likely among college graduates than those without college degrees [12]. Abuse of illicit drugs and alcohol are linked to several health issues including hepatitis C, HIV/AIDS, and cardiovascular complications [13, 14]. Substance use also contributes to a significant economic cost due to crime, lost work productivity, and health care needs [15]. Additionally, HIV infection, which disproportionately affects African Americans and Hispanics/Latinos, may have mixed effects on barriers to care [16, 17]. HIV infection may lead to better access to care through broadened eligibility for medical care but also add to existing barriers to care through disability and financial burden caused by the disease [2, 18]. The diverse range of problems faced by marginalized populations suggests a need to carefully examine how the intersections of substance use and HIV status are associated with perceived access and barriers to care. Additionally, previous research has suggested a positive impact of social support on health outcomes and behaviors [19]. It is important to assess whether the presence of social support may affect the association between substance use, HIV status, and access and barriers to care in marginalized populations.

The National Institute on Drug Abuse funded “Seek, Test, Treat, Retain” (STTR) data harmonization initiative provides a unique opportunity to examine populations who are typically difficult to reach because of marginalization due to race/ethnicity, substance use, incarceration history, and HIV status. The STTR consortium consists of twenty-three observational studies and randomized controlled trials that assessed ways to improve HIV testing outreach to high-risk populations, and for those known to be HIV-infected, ART initiation and retention in long-term care and treatment [20, 21]. The current analyses included baseline data from six STTR studies that collected information related to perceived access and barriers to health care, using standardized questionnaires. Participants were identified as vulnerable populations living in urban settings, because they either had recent criminal justice involvement or received care at safety-net settings and were at risk for or already had HIV infection and/or substance abuse issues.

The current study harmonized data among consortium studies in order to assess various kinds of barriers to care faced by subgroups of a marginalized population as well as differences in perceived access and barriers to care by substance use and HIV status and the role of social support in these associations. The overall aim was to gain insight into issues related to access to care that could be useful for planning interventions to improve access to and quality of care for marginalized populations.

## Methods

### Study settings

The current analyses used baseline data from six studies (BCAP/BCU, C4C, FIRST, STAR, BRIGHT2, STTCOIP-Prison) in the STTR consortium [21–23]. These six studies were selected based on availability of data on measures related to access and barriers to care and drug and alcohol use. The study participants were all from urban settings in the United States (New York, San Francisco, Baltimore, and Chicago). Assessments in these studies were completed between April 2011 and November 2015. The summary of the studies included in the current analyses is shown in Additional file 1: Table S6.

### Measurements

#### Exposure variables and covariates

All included studies administered structured questionnaires to collect data on self-reported demographic characteristics (age, gender, race/ethnicity, and education), social support, and substance use patterns. Those who identified as transgender were not included in the analysis as they may face unique challenges but the sample size was too small to conduct meaningful analyses (*n* = 17). Education level was categorized into 3 groups based on the highest degrees obtained: Less than high school, high school diploma/some college education, and college degrees and above. The Alcohol Use Disorder Identification Test (AUDIT) was used to identify hazardous drinking patterns (≥7 for women; ≥8 for men) over the past 6 months (C4C, STTCOIP-Prison) or the past year (BCAP/BCU, FIRST, STAR) [24, 25]. Illicit drug use was defined as recent use of any illicit drugs in the past 30 days (BCAP/BCU, FIRST, STAR), 90 days (BRIGHT2), and 6 months (C4C, STTCOIP-Prison). The definition of illicit drugs does not include marijuana but both illicit drug users and non-drug users included some marijuana users. Illicit drug use included all routes of administration. HIV status was determined by serological testing conducted within each study (BCAP, BCU, STAR, C4C, BRIGHT2) or medical records (FIRST and STT-COIP Prison). Social support score was based on summated points from 5 questions related to social support developed from previous tools designed to assess social support among HIV-positive individuals (Additional file 1: Table S7), which asked how often each of the kinds of support was available to the participants over the past 4 weeks if they needed it and the answers ranged from 1 to 5 (“none of the time” to “all of the time”) [26, 27].

#### Outcome measures of perceived access and barriers to care

Thirteen questions addressing issues related to access and barriers to care were constructed for the STTR initiative based on previous studies, as listed in Tables 2 and 3 [2, 3, 28]. There were 6 items in the domain of perceived access to care and 7 items for barriers to care. Items related to perceived access to care were asked on a Likert scale (1–5; strongly agree to strongly disagree) while barriers to care required binary answers (yes/no). For perceived access to care, all responses were assigned values where higher values indicated better conditions to align the direction of the scales. For example, in the question “If I need medical care, I can get admitted without any trouble”, those strongly agreeing to the statement were given 5 while those strongly disagreeing with “It is hard for me to get medical care in an emergency” were also given 5. For barriers to care, lower scores (*i.e.* having fewer barriers) indicated better conditions. For descriptive comparison, those who had scores 1 or 2 for each question in access to care and 1 for barriers to care were categorized as having difficulties.

### Statistical analysis

Descriptive statistics were calculated for the total population, non-drug users (DU-), drug users (DU+), non-hazardous drinkers/abstainers (HD-), and hazardous drinkers (HD+). The proportion of participants reporting barriers for each individual item was calculated for comparison by illicit drug use status, hazardous drinking patterns, gender, and HIV status. Two-proportions z-tests were performed to compare the proportions reporting worse access of care or barriers to care among DU-/HD- to each of the substance use groups (DU-/HD+, DU+/HD-, DU+/HD+). For comparison of mean domain scores among subgroups, a standardized score for each domain was calculated by summating points from individual items and standardizing them by subtracting the mean from the summated points and dividing by the standard deviation [29]. These standardized scores were plotted in boxplots in four subgroups defined by substance use pattern (illicit drug use or hazardous drinking) and HIV status. Jitter plots were overlaid to show the density and distribution of the score for each subgroup. Mean standardized scores were also shown on the graph. The Welch two-sample t-test was used to compare mean values among subgroups.

For multivariable linear regression analyses, the raw score for each domain (*i.e.* summated points from individual questions within each domain) was used for outcomes. The associations of these scores with drug use and hazardous drinking were examined in multivariable regression models adjusting for age, gender, race/ethnicity, and social support. All models were stratified by HIV status. Multilevel regression models were also fit to test study-level differences but the intraclass correlations for the study level were small (0.01 to 0.15), and therefore simple regression models were chosen. Mediation analyses were also performed to see how much of the association between substance use and access and barriers to care could be explained by social support, if social support were a mediator between substance use and perceived access and barriers to care. Separate analyses were performed for both types of substance use (drug use and hazardous drinking), using the following models: 1) mediator conditioned on exposure (social support on substance use, adjusting for age, gender, race/ethnicity, the other type of substance use) and 2) outcome conditioned on exposure and mediator (score from each domain on drug use, hazardous drinking, and social support, adjusting for age, gender, and race/ethnicity). The mediation analysis was only retained in HIV-negative participants as the association between the mediator and exposure was not observed in HIV-positive participants. All analyses were performed in R (Version 3.0.2).

## Results

### Descriptive statistics

#### Characteristics of the participants

A total of 6387 participants were included in this study, among whom 71% were male and most were from minority populations (57% Blacks/African Americans and 39% Hispanics/Latinos) (Table 1). The median age was 42 years old (range 18–75). Educational attainment was low; 35% had less than high school degree in comparison to 11.6% in the general population in the U.S. in 2015 [30]. Among HIV-positive participants, 56% reported having recently used illicit drugs, considerably higher than the HIV-negative participants (17%). Higher proportions of Hispanic/Latino participants reported having used illicit drugs recently (30%) and drunk hazardously (38%) than African American participants (15% and 26% respectively). Thirty-five to 48 % of the population reported not having someone to help buy medicines, help with transportation, or provide financial assistance when needed (Additional file 1: Table S7). Greater proportions of HIV-positive participants and men reported having less support on all items related to social support than HIV-negative participants and women respectively (Additional file 1: Table S7).

**Table 1:** Characteristics of the study participants

	All	DU-^a^	DU+^a^	HD-	HD+
Age (years) mean (sd)	*n* = 6387	4984	1403	4261	1889
	39.6(12.2)	38.3(12.4)	43.8(10.5)	38.8(12.6)	41.2 (11.3)
Gender *n* (%)					
Men	4557 (71.3)	3316 (66.5)	1241 (88.5)	2824 (66.3)	1567 (83.0)
Women	1830 (28.7)	1668 (33.5)	162 (11.5)	1437 (33.7)	322 (17.0)
Race n (%)					
Hispanic/Latino	2475 (38.8)	1736 (34.8)	739 (52.7)	1514 (35.5)	931 (49.3)
Black/African American	3651 (57.2)	3118 (62.6)	533 (38)	2556 (60)	898 (47.5)
White/Asian	107 (1.7)	24 (0.5)	83 (5.9)	83 (1.9)	21 (1.1)
Other	154 (2.4)	106 (2.1)	48 (3.4)	108 (2.5)	39 (2.1)
HIV status n (%)					
Negative	5509 (86.3)	4594 (92.2)	915 (65.2)	3721 (87.3)	1663 (88.0)
Positive	878 (13.7)	390 (7.8)	488 (34.8)	540 (12.7)	226 (12.0)
Hazardous drinking^b^ n (%)					
No	4261 (69.3)	3531 (73.7)	730 (53.7)		
Yes	1889 (30.7)	1259 (26.3)	630 (46.3)		
Education n (%)					
Less than high school	2217 (34.8)	1755 (35.3)	462 (33)	1439 (33.8)	691 (36.6)
High school/some college	3901 (61.2)	3034 (61)	867 (61.8)	2637 (62)	1129 (59.9)
College degree or higher	256 (4)	183 (3.7)	73 (5.2)	178 (4.2)	66 (3.5)
Social support^c^ n (%)					
No	1763 (35.3)	1317 (33)	446 (44.6)	1107 (32.6)	612 (41.5)
Yes	3232 (64.7)	2679 (67)	553 (55.4)	2290 (67.4)	864 (58.5)

All participants have data on gender (men/women), race (black/African American, Hispanic/Latino, White/Asian, Other), and HIV status (positive/negative) and answered all questions within either or both domains of barriers to care (perceived access to care; barriers to care). Those who refused to answer race/ethnicity questions were categorized under “Other” (*n* = 22). Those who identified as transgender were not included in the analyses

Percentages are calculated for each characteristic (i.e. Race) within each substance use group

*DU* illicit drug users, *HD* hazardous drinkers

^a^Some of the *DU-* and *DU+* may concurrently use alcohol and marijuana

^b^*Hazardous drinking* is defined by AUDIT score ≥ 8 (male) and 7 (female)

^c^*Social support (yes*) is assigned to participants who reported having support in the majority of the items on the social support questionnaire (≥3 items)

#### Access to care

**Fig. 1:**
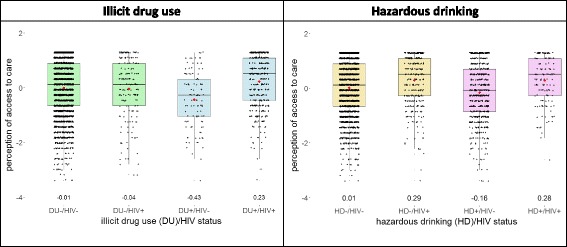
Distribution of perceived access to care scores by HIV status and substance use patterns. Each box represents the 1st to 3rd quartiles with the median line. The jitter plots show the standardized score distributions. The mean values are marked as red dots and the values are noted under each boxplot. The whiskers indicated the lowest and highest values within 1.5 times 1st and 3rd quartile values. For this domain, higher standardized scores indicate better conditions

Nearly one-third (29%) of the study population reported that they sometimes go without the medical care they need because it is too expensive, which is similar to the general population (27%) (Table 2) [31]. More than one quarter of the study population reported not having easy access to medical specialists. Men perceived having worse access to care than women (Additional file 1: Table S8). Difficulties with medical expense and access to specialists were more commonly reported among the HIV-negative participants than HIV-positive participants (Additional file 1: Table S8). As shown in Fig. 1, HIV-positive illicit drug users (DU+) participants had overall better perceived access to care than HIV-negative DU+ participants (mean ± standard deviation (sd): 0.23 ± 0.99 and − 0.43 ± 1.01 respectively; p for the difference < 0.001). The difference in overall perceived access to care between HIV-negative and HIV-positive participants was much larger among DU+ than non-drug users (DU-) (Fig. 1). HIV-positive participants had higher mean scores than HIV-negative participants in both HD- and HD+ groups.

**Table 2:** Proportions reporting difficulties in access to care by substance use patterns

	All		Substance use patterns							
			no DU and no HD^a^		DU only		HD only		DU and HD	
Perceived Access to Care	*n*	%	*n*	%	*n*	%	*n*	%	*n*	%
If I need medical care, I can get admitted without any trouble (% disagree)	3548	11.4	2325	11.6	319	11	620	11	152	7.2
It is hard for me to get medical care in an emergency (% agree)	3554	19.8	2329	18.8	319	19.4	620	22.7^*^	152	21.1
I have easy access to the medical specialists that I need. (% disagree)	3546	26.9	2326	26.4	319	16.3^**^	619	30.4	152	18.4^*^
I am able to get medical care whenever I need it (% disagree)	3557	12.7	2332	11.1	319	12.5	622	14	152	14.5
Places where I can get medical care are very conveniently located (% disagree)	3552	13.7	2328	12	319	17.2^**^	621	14.7	152	19.1^*^
Sometimes I go without the medical care I need because it is too expensive (% agree)	3551	28.6	2326	27.7	319	25.7	622	31.7	152	23

Two-proportions z-test was performed between no DU or HD group and each of the substance use groups (DU only, HD only, DU and HD). * indicates *p* < 0.05 and ** indicates *p* < 0.01

*DU* illicit drug users, *HD* hazardous drinkers

^a^This group includes participants who consume alcohol but do not drink hazardously

#### Barriers to care

**Fig. 2:**
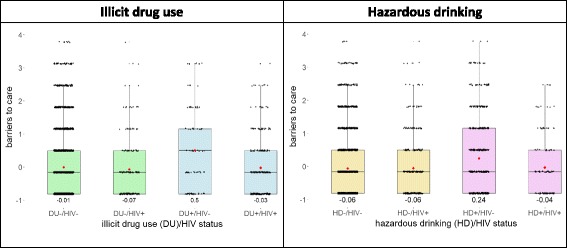
Distribution of perceived barriers to care scores by HIV status and substance use patterns. Each box represents the 1st to 3rd quartiles with the median line. The jitter plots show the standardized score distributions. The mean values are marked as red dots and the values are noted under each boxplot. The whiskers indicated the lowest and highest values within 1.5 times 1st and 3rd quartile values. For this domain, lower standardized scores indicate better conditions

Seventeen percent of the study participants were uninsured. DU+ and HD+ generally reported more barriers to care than DU-/HD- (Table 3). More than 18% of substance users reported having been treated poorly at a clinic in the past in comparison to 14% for DU-/HD-. Inability to pay for medical care and lack of a means of transportation were common barriers reported in the study population. Higher proportion of the DU+/HD+ participants (21%) reported mistrust in doctors than DU-/HD-, DU+/HD-, and DU-/HD+ (11%, 17%, and 15% respectively). Men reported more barriers to care than women on all items, including the lack of insurance (19.2% in men and 9.7% in women) (Additional file 1: Table S8). There were similar trends for overall barriers to care in DU and HD (Fig. 2): HIV-negative DU+ and HD+ groups had higher mean standardized scores for barriers to care than the other subgroups. The differences in mean standardized scores by HIV status were small among non-substance users. HIV-positive HD+ and DU+ groups had similar mean standardized scores as HD- and DU-.

**Table 3:** Proportions reporting barriers to care by substance use patterns

	All		Substance use patterns							
			no DU and no HD^a^		DU only		HD only		DU and HD	
Barriers to care: “Think of the last time you did not get the medical treatment recommended for you”	*n*	%	*n*	%	*n*	%	*n*	%	*n*	%
I was unable to pay for medical care (% agree)	3448	27.9	2388	25.7	263	27^**^	632	34.8^**^	126	32.5^**^
I did not have transportation to medical care (% agree)	3476	26.4	2386	23	287	34.5^**^	632	32.1^**^	134	42.5^**^
The clinic’s hours of operation were inconvenient for me (% agree)	3474	18.2	2386	16	287	22.3^**^	631	21.6^**^	134	27.6^**^
I did not have child care (% agree)	3444	10.2	2361	9.8	287	8.7	627	11.3	131	10.7
I was treated poorly at a clinic in the past (% agree)	3481	16	2390	13.7	287	17.8^**^	632	21.4^**^	134	24.6^**^
I do not trust doctors (% agree)	3468	12.9	2383	11.3	286	17.1^**^	628	14.6^*^	134	20.9^**^
Uninsured (%)	6342	16.5	3512	15.9	720	12.9	1254	17.1	622	20.4^**^

Two-proportions z-test was performed between no DU or HD group and each of the substance use groups (DU only, HD only, DU and HD). * indicates *p* < 0.05 and ** indicates *p* < 0.01

*DU* illicit drug use, *HD* hazardous drinking

^a^This group includes participants who consume alcohol but do not drink hazardously

### Multivariable regression models

Illicit drug use was associated with worse perceived access to care in HIV-negative participants (β: − 1.42 (95% confidence interval (CI): − 2.38 to − 0.45)), adjusting for age, gender, race/ethnicity, and social support (Table 4).

**Table 4:** Multivariable regression models assessing association of drug use and hazardous drinking with perceived access to care and barriers to care, stratified by HIV status

	Perceived access to care^a^		Barriers to care^a^	
Better condition	Higher score		Lower score	
	β (95%CI)	*p*	β (95%CI)	*s*
HIV-negative	*n* = 2895		*n* = 2832	
Illicit drug use (yes)	−1.42 (−2.38 to −0.45)	< 0.01	0.49 (0.19 to 0.79)	< 0.01
Hazardous drinking (yes)	−0.34 (−0.78 to 0.11)	0.14	0.31 (0.18 to 0.45)	< 0.01
Social support	0.18 (0.15 to 0.22)	< 0.01	− 0.05 (− 0.06 to − 0.04)	< 0.01
HIV-positive	*n* = 263		*n* = 253	
Illicit drug use (yes)	1.89 (0.56 to 3.22)	0.01	− 0.01 (− 0.39 to 0.38)	0.97
Hazardous drinking (yes)	0.18 (− 0.96 to 1.32)	0.75	−0.03 (− 0.43 to 0.37)	0.86
Social support^a^	0.18 (0.09 to 0.28)	< 0.01	−0.02 (− 0.05 to 0.01)	0.15

All models were adjusted for age, gender, and race/ethnicity

^a^Scores were based on summation of points from individual questions within each domain (perceived access to care: 6 questions; barriers to care: 7; social support: 5). The domain score ranges were: perceived access to care 5–30 points; barriers to care 0–7 points; social support 5–25 points

In HIV-negative participants, both illicit drug use and hazardous drinking were strongly associated with greater barriers to care (β: 0.49 (95% CI: 0.19 to 0.79) and 0.31 (0.18 to 0.45) respectively). However, there was no evidence for association between substance use and barriers to care in HIV-positive participants. There were no interactions between social support and DU or HD in these models. Social support was positively associated with perceived access to care and inversely with barriers to care in HIV-negative participants.

### Mediation analysis

We also explored a possibility of mediation by social support in the association between substance use and perceived access and barriers to care. In HIV-positive participants, there was no strong evidence for the association between social support and DU/HD, and therefore mediation analysis was not undertaken (Additional file 1: Table S9). In HIV-negative participants, there was evidence of mediation by social support between hazardous drinking and both perceived access to care (ACME: − 0.24 (− 0.35 to − 0.15) *p* < 0.01; proportion mediated 0.42 (0.2 to 1.61 *p* = 0.01) and barriers to care (ACME: 0.06 (0.04 to 0.09) *p* < 0.01; proportion mediated 0.16 (0.09 to 0.29) *p* < 0.01) (Table 5); however, the mediation effect was not apparent in the association between drug use and measures of perceived access and barriers to care.

**Table 5:** Mediation effects by social support in the association of drug use and hazardous drinking with perceived barriers to care and barriers to care in HIV-negative participants

	Perceived access to care		Barriers to care	
Better condition	Higher score		Lower score	
Illicit drug use				
ACME	−0.17 (− 0.35 to 0)	0.06	0.05 (− 0.01 to 0.1)	0.07
ADE	− 1.42 (− 2.33 to − 0.37)	< 0.01	0.49 (0.13 to 0.86)	0.02
Total effects	− 1.59 (− 2.58 to − 0.53)	< 0.01	0.54 (0.18 to 0.92)	0.01
Proportion mediated	0.11 (0 to 0.32)	0.07	0.09 (− 0.02 to 0.3)	0.08
Hazardous drinking				
ACME	−0.24 (− 0.35 to − 0.15)	< 0.01	0.06 (0.04 to 0.09)	< 0.01
ADE	−0.34 (− 0.78 to 0.11)	0.18	0.31 (0.17 to 0.45)	< 0.01
Total effects	−0.58 (− 1.03 to − 0.13)	0.01	0.38 (0.23 to 0.51)	< 0.01
Proportion mediated	0.42 (0.2 to 1.61)	0.01	0.16 (0.09 to 0.29)	< 0.01

All models were adjusted for age, gender, race/ethnicity

*ACME* Average Causal Mediation Effects, *ADE* Average direct effect

## Discussion

The current study found that recent illicit drug use and hazardous drinking as well as low social support were associated with greater barriers to care in the HIV-negative participants. Among HIV-positive participants, this association was less prominent. Financial burden of care, logistical difficulties in accessing care, and lack of social support were commonly reported in this marginalized population.

### Comparison with previous studies

Previous research has shown that the intersection of substance use and HIV can present significant challenges for both the patients and health care systems [32]. Illicit drug users may be less motivated to use routine health care to avoid inquiry and monitoring of their drug use while at the same time, they may be more likely to present for emergency room visits and hospital care [33]. The underlying mechanisms for low usage of routine care among illicit drug users include comorbid psychiatric conditions such as depression and bipolar disorder as well as mistrust in health care professionals and systems [32, 34, 35]. Alcohol abuse is also associated with a number of psychiatric comorbidities, which may contribute to worse access and greater barriers to care [36]. Illicit drug users are also more likely to have alcohol disorder [37]. We observed this association in the current study, where the prevalence of hazardous drinking was much higher among illicit drug users than non-drug users (46% vs 26%). It is important to understand how each condition, as well as combinations of these conditions, is associated with barriers to care. In our study, the participants who were both using illicit drugs and drinking hazardously reported having more logistical issues, less trust in health care, and less social support than those who only used illicit drugs or drank hazardously, although we did not observe interaction effects between illicit drug use and hazardous drinking in our multivariable models.

In our study population, HIV infection was common among illicit drug users. In the United States, the HIV epidemic disproportionately affects impoverished individuals in urban settings and minority populations [16, 17, 38]. Even though HIV infection greatly increases the need for receiving continuous care, engagement in and adherence to HIV care remains a major public health challenge; in 2014, only 58% of HIV-positive people achieved viral suppression [39]. Studies have reported poor adherence to HIV care among drug users [40–42]. The mechanisms underlying the association between HIV infection and health care are complex; on the one hand, HIV-infected individuals may experience other comorbidities as well as greater stigma and financial difficulties, which may prevent them from attending clinics even if they are available. However, they also have greater needs for regular treatment, which may motivate them to seek routine care, and there is also a strong public infrastructure to support the care of HIV-infected individuals in the U.S [43]. In our study, we saw that HIV-positive participants had, on average, a better aggregate score of perceived access to care, suggesting that some factors associated with being infected with HIV (*i.e.* strong public clinic infrastructure, personal health needs, programs to link HIV-infected individuals to care, and AIDS drug assistance programs) may potentially counteract adverse conditions associated with HIV infection. In addition to having more comprehensive and resourced care, HIV providers may be less stigmatizing and discriminatory towards people who use drugs than providers who care for HIV-negative patients, since HIV providers often encounter substance use in their patients. This may partially explain better perceived access to care we saw among HIV-positive substance users, who may receive additional referrals from their providers for substance use treatment.

We also found that social support among HIV-positive participants was lower than HIV-negative participants. Social support is thought to alter biological processes and affect health outcomes through its influence on behavioral and psychological processes [44]. Social support may influence HIV disease progression physiologically and psychologically by affecting immune systems as well as providing functional support to facilitate better adherence to treatment [5, 44–50]. Additionally, HIV infection may make patients withdraw from social networks, resulting in a negative feedback loop between social support and the disease status [51, 52]. Likewise, substance users may also experience this kind of isolation. In our study, we examined social support as a confounder and also explored the possibility of its role as a mediator in the association between substance use and access to care. In the multivariable models, substance use was only associated with social support among HIV-negative participants. This may be because the level of social support is already so low among HIV-positive participants that drug use and hazardous drinking may not add discernable effects. It is also important to note that there are other types of social support that may contribute to better access to care among HIV-positive participants beyond what the current study measured, which focused on support by families and friends. Future studies are needed to better understand the associations between different types of social support and HIV care continuum.

The study population consisted largely of marginalized racial/ethnic minority groups (*i.e*. African Americans or Hispanic/Latinos living in areas with a high prevalence of HIV infection, illicit drugs, and criminal history). Research has demonstrated that minority groups receive lower-quality care and have lower trust in health care systems than Whites [3, 4]. While socioeconomic factors like poverty, insurance coverage, and education partially explain the association between access to care and race/ethnicity, there may also be an independent effect of race/ethnicity on access to care [53]. There may also be variability in types of barriers to care among minority groups. For instance, a qualitative study found that Hispanics/Latinos may have more linguistic barriers while African Americans may have lower trust in health care professionals [54]. In our study, Hispanics/Latinos generally reported greater barriers to care and lower social support than Blacks/African Americans. However, the racial/ethnic differences we saw in our study were generally not strong, which is likely due to the fact that we are comparing two minority groups rather than against a sizable privileged group.

We also saw that men in our study population perceived having worse access to care than women. In this study population, the proportion of uninsured men was greater (19%) than the general population (13%) as well [55]. Previous research that used the same instrument for perceived access to care have shown variable results for gender differences in perceived access to care [56, 57]. One possible explanation for this variability is that gender differences in perceived access to care may vary between subpopulations. There is a need to further assess which specific context may contribute to gender differences in perceived access to care in marginalized populations and how that is associated with health care utilization.

### Public health implications

Given these findings in the current study, we may need to consider building additional infrastructure to improve access to care for marginalized populations who are at risk but not infected with HIV as care for these individuals are not well designed to meet their needs that often are similar to those for HIV-positive individuals. In the mediation analysis, there was some evidence that a large proportion of the association between hazardous drinking and perceived access and barriers to care may be mediated through social support in HIV-negative individuals. This analysis cannot confirm whether social support is acting as a confounder or a mediator but they do suggest a need for future studies to elucidate the role of social support among substance users. If social support is indeed a mediator of this association, our finding has an important policy implication, since increasing social support could potentially contribute to reduction of barriers to care in marginalized populations. Several studies have examined effectiveness of social support interventions and found that types of social support needed may be highly context-dependent [58]. To add to this complexity, specific types of barriers to care as well as subgroups of marginalized populations who experience most difficulties in access to care may change over time with political climate and cultural shift. There is a need to monitor such changes and build dynamic infrastructure that can cope with variable difficulties faced by marginalized populations.

### Limitations

The study used cross-sectional data and therefore, we cannot infer causality from our findings. Substance use and barriers to care may mutually affect each other. The relationships could well be bidirectional; for example, substance use may lead to experiencing greater barriers to care through loss of motivation and productivity, financial burden, and social isolation; likewise, experiencing these difficulties may lead people to initiate or increase substance use, especially for difficult conditions like chronic pain. If the mechanisms underlying the associations we saw between substance use and barriers to care are bidirectional, they can mutually result in worse health outcomes, where the problems are self-reinforcing. To break these issues apart would require careful longitudinal studies. Substance use was based on self-report data and may be affected by cognitive bias. Our sample size for HIV-positive participants was smaller than the HIV-negative participants, which makes it difficult to make conclusive remarks about the lack of association between substance use and barriers to care in HIV-positive participants. HIV-negative participants came from two studies in New York and future studies from other cities are necessary to assess generalizability of our findings. Furthermore, future studies in rural settings are needed to understand any differences in needs among urban and rural residents.

We also did not have information on how these reported barriers may be associated with health care utilization or health outcomes, both of which are important elements to consider. Not all types of potential barriers - for instance, linguistic and cultural barriers to care [4] or food insecurity [59] - could be examined due to insufficient data availability. The data were taken from study populations in various locations although all of these studies were conducted in major urban areas in the United States and aimed to recruit marginalized populations, providing some consistency in participant characteristics.

## Conclusions

Drug abuse and hazardous drinking present challenges to health care access in marginalized populations. The participants in this study experienced high degrees of barriers to care, especially in terms of financial burden, logistic difficulties in accessing care, and lack of social support. Our findings suggest the needs to strengthen infrastructural and social support for marginalized populations regardless of HIV status.

## Additional file

Additional file 1: Supplemetal **Tables S6** (Summary of the STTR studies included in this study), **S7** (Comparison of proportions reporting no or low social support by gender and HIV status), **S8** (Comparison of proportions reporting low perceived access to care and greater barriers to care by gender and HIV status), **S9** (Multivariable regression models assessing association of drug use and hazardous drinking with the mediator variable (social support), stratified by HIV status). (DOCX 23 kb)

## Abbreviations

CI: Confidence interval
DU: Illicit drug use
HD: Hazardous drinking
HIV: Human immunodeficiency virus
STTR: Seek, test, treat, retain
