# Supplementary material for: Perceived access and barriers to care among illicit drug users and hazardous drinkers: findings from the Seek, Test, Treat, and Retain data harmonization initiative (STTR)
Source: BMC Public Health. 2018 Mar 20;18:366. doi: 10.1186/s12889-018-5291-2 (PMC5859651; doi:10.1186/s12889-018-5291-2)
Supplement: Supplementary file 1 — Supplemetal Tables S6 (Summary of the STTR studies included in this study), S7 (Comparison of proportions reporting no or low social support by gender and HIV status), S8 (Comparison of proportions reporting low perceived access to care and greater barriers to care by gender and HIV status), S9 (Multivariable regression models assessing association of drug use and hazardous drinking with the mediator variable (social support), stratified by HIV status). (DOCX 23 kb) [file 12889_2018_5291_MOESM1_ESM.docx]

## Supplemental Material:

## Table 6: Summary of the STTR studies included in this study.

| **study** | **location** | **men/**  **women (n)** | **HIV+ (%)** | **Study population** | **recruitment methods** | **barriers to care data availability** | **social support**  **data^1^** |
| --- | --- | --- | --- | --- | --- | --- | --- |
| [BCAP](https://clinicaltrials.gov/ct2/show/NCT01607541)/[BCU](https://clinicaltrials.gov/ct2/show/NCT02421159) | New York | 2260/  1708 | 4% | Heterosexuals living in areas at high risk of HIV | Respondent-driven sampling and venue-based sampling | Access Barriers | Available |
| [FIRST](https://clinicaltrials.gov/ct2/show/NCT01376570) | New York | 98/51 | 100% | HIV-infected drug users receiving drug and HIV treatment who have suboptimal drug treatment (using cocaine or opiods while on opioid agonist treatment) and suboptimal HIV treatment (detectable HIV viral load while prescribed highly active antiretroviral therapy) | Active (i.e. via medical staff, letters) and passive (i.e. flyers, advertisements, word-of-mouth) recruitment strategies. | Access Barriers | Available |
| [STAR](https://clinicaltrials.gov/ct2/show/NCT01790360) | New York | 1853/  11 | 100% | HIV-infected AA substance-using transgender women or men who have sex with men identified by respondent driven sampling | Respondent-driven sampling | Access Barriers | Available |
| [C4C](https://www.ncbi.nlm.nih.gov/pubmed/25551175) | San Francisco | 190/30 | 100% | HIV-infected patients new to clinic or with a history of poor retention in clinic care. | Recruited through an HIV clinic | Access Barriers | Not available |
| [BRIGHT2](https://www.ncbi.nlm.nih.gov/pmc/articles/PMC3674156/) | Baltimore | 78/22 | 100% | HIV-infected probationers and parolees. | Flyers distributed through informational forums and by probation officers. | Access | Available |
| [STT-COIP Prison](https://clinicaltrials.gov/ct2/show/NCT01852877) | Chicago | 78/8 | 100% | HIV-infected persons who have recently been released from jail/prison. | Prisoners are recruited by telemedicine case manager 1-3 months prior to scheduled release. | Access | Available |

1. Based on the standardized questionnaire shown in Table 7.

## Table 7: Comparison of proportions reporting no or low social support by gender and HIV status.

|  | | | **gender** | | | | **HIV status** | |
| --- | --- | --- | --- | --- | --- | --- | --- | --- |
|  | | | **men** | | **women** | | **negative** | **positive** |
| **Social Support: “How often was each of the following kinds of support (over the past 4 weeks) available to you if you need it?”** | **all** | n=3605 | | 1404 | | 4371 | | 638 |
| **Someone to love and make you feel wanted? (% No)** | 18.9 | 21.3 | | 12.9 | | 18.6 | | 21 |
| **Someone to help with daily chores (child care, buying food, preparing meals) if you were sick? (% No)** | 34.5 | 37.4 | | 27.1 | | 33.7 | | 39.8 |
| **Someone to help you buy medicines? (% No)** | 47.5 | 50.6 | | 39.6 | | 46 | | 57.9 |
| **Someone to help with transportation? (% No)** | 40.4 | 42.5 | | 35.1 | | 39.4 | | 47.2 |
| **Someone to give you money if you needed it? (% No)** | 37.7 | 38.7 | | 35 | | 37 | | 42 |

No/low social support was defined as having the following social support “none of the time” or “a little of the time”.

## Table 8: Comparison of proportions reporting low perceived access to care and greater barriers to care by gender and HIV status.

|  | **gender** | | **HIV status** | |
| --- | --- | --- | --- | --- |
|  | **men** | **women** | **negative** | **positive** |
| **Perceived Access to Care** | n=2197 | 1351 | 2964 | 584 |
| **If I need medical care, I can get admitted without any trouble (% disagree)** | 12.4 | 9.8 | 11.9 | 9.2 |
| **It is hard for me to get medical care in an emergency (% agree)** | 20.4 | 18.9 | 19.6 | 21.2 |
| **I have easy access to the medical specialists that I need. (% disagree)** | 28.4 | 24.4 | 27.9 | 21.7 |
| **I am able to get medical care whenever I need it (% disagree)** | 14.3 | 10.2 | 12.2 | 15.4 |
| **Places where I can get medical care are very conveniently located (% disagree)** | 15.2 | 11.4 | 13.2 | 16.6 |
| **Sometimes I go without the medical care I need because it is too expensive (% agree)** | 31.5 | 23.9 | 29.3 | 25 |
| **Barriers to care: “Think of the last time you did not get the medical treatment recommended for you”** | 2147 | 1301 | 2963 | 485 |
| **I was unable to pay for medical care (% agree)** | 31.6 | 21.7 | 28.9 | 21.9 |
| **I did not have transportation to medical care (% agree)** | 27.8 | 24.2 | 25.6 | 31 |
| **The clinic’s hours of operation were inconvenient for me (% agree)** | 19.3 | 16.3 | 17.9 | 19.6 |
| **I did not have child care (% agree)** | 10.3 | 9.9 | 10.8 | 6.4 |
| **I was treated poorly at a clinic in the past (% agree)** | 17.6 | 13.4 | 15.9 | 16.3 |
| **I do not trust doctors (% agree)** | 13.4 | 12.1 | 12.3 | 16 |
| **Uninsured** | n=4516 | 1826 | 5476 | 866 |
|  | 19.2 | 9.7 | 16.7 | 15.1 |

## Table 9: Multivariable regression models assessing association of drug use and hazardous drinking with the mediator variable (social support), stratified by HIV status.

|  | **Social support** | |
| --- | --- | --- |
| **Better condition** | **Higher score** | |
|  | **β (95%CI)** | **p** |
| **HIV-negative** | **n = 4348** |  |
| **Illicit drug use (yes)** | -1.03 ( -1.52 to -0.55 ) | <0.01 |
| **Hazardous drinking (yes)** | -1.04 ( -1.42 to -0.67 ) | <0.01 |
| **HIV-positive** | **n = 525** |  |
| **Illicit drug use (yes)** | -0.57 (-1.61 to 0.48) | 0.29 |
| **Hazardous drinking (yes)** | -0.56 (-1.66 to 0.53) | 0.31 |

The models were adjusted for age, gender, and race/ethnicity.
